# Fulminant Influenza A Myocarditis in a Healthy Young Adult Salvaged With Biventricular Impella Support: A Case Report

**DOI:** 10.7759/cureus.89322

**Published:** 2025-08-04

**Authors:** Ahmad Jalil, Fatima Rajab, Maheen Zaidi

**Affiliations:** 1 Internal Medicine, Baptist Memorial Hospital-North Mississippi, Oxford, USA; 2 Internal Medicine, King Edward Medical University, Lahore, PAK

**Keywords:** fulminant myocarditis, impella device, influenza a infection, interventional cardiology, young adult male

## Abstract

Fulminant myocarditis is a rare but life-threatening complication of influenza A infection that can result in acute biventricular failure leading to cardiogenic shock. Here, we present the case of a young patient who developed acute bilateral heart failure secondary to influenza A and was successfully stabilized using both right and left-sided Impella devices. This case highlights the critical role of early and aggressive mechanical circulatory support (MCS) in managing fulminant myocarditis and emphasizes the utility of Impella in cases of severe cardiac dysfunction. Our patient showed a remarkable recovery, with significant improvement in ejection fraction within days of initiating MCS. Notably, a strong family history of myocarditis was present, raising the possibility of a genetic predisposition to developing fulminant myocarditis in response to viral infections. This case underscores the potential for serious and potentially fatal cardiogenic complications of a common viral illness in genetically susceptible individuals.

## Introduction

Influenza virus remains a major contributor to respiratory tract infections worldwide, with an incidence of approximately 8.3% across all age groups in the United States [1]. While most cases present with self-limiting upper respiratory symptoms, influenza can occasionally result in severe systemic complications. One such underrecognized but potentially devastating complication is myocarditis, which is estimated to occur in up to 10% of influenza infections [2]. The clinical spectrum of influenza-related myocarditis ranges from subclinical myocardial inflammation to fulminant myocarditis with rapid hemodynamic collapse. In its most severe form, fulminant myocarditis can lead to acute biventricular failure and cardiogenic shock, necessitating emergent hemodynamic support.

Mechanical circulatory support (MCS) plays a crucial role in stabilizing these critically ill patients. Several MCS modalities have been utilized in this setting, including intra-aortic balloon pumps, extracorporeal membrane oxygenation, and percutaneous ventricular assist devices such as the Impella system [3,4]. The selection of an appropriate support strategy depends on the severity of ventricular dysfunction, presence of biventricular involvement, and institutional expertise.

Here, we present a compelling case of a previously healthy 24-year-old male who developed fulminant myocarditis secondary to influenza A infection, resulting in acute cardiogenic shock and biventricular failure. He was successfully stabilized using bilateral percutaneous ventricular assist devices, Impella CP for left ventricular support, and Impella RP for right ventricular support. This case highlights the importance of early recognition of cardiac involvement in influenza infections and the lifesaving potential of timely initiation of advanced mechanical support.

## Case presentation

A 24-year-old male with no significant past medical history or known cardiovascular risk factors presented to the hospital with altered mental status. He had been experiencing flu-like symptoms for the past few days before the presentation. He was not taking any medications and had no history of substance abuse. On arrival, his vital signs included a blood pressure of 118/57 mmHg, a respiratory rate of 30 breaths per minute, a heart rate of 167 beats per minute, oxygen saturation of 96%, and a temperature of 100.9°F. On physical examination, he appeared pale and diaphoretic and was tachycardic. Laboratory values are presented in Table 1.

**Table 1:** Laboratory values of our patient on day one.

Laboratory parameter	Value	Reference range
pH	7.37	7.35–7.45
pCO_2_	19 mmHg	35–45 mmHg
pO_2_	71 mmHg	80–100 mmHg
White blood cells	11,100 cells/µL	4,500–11,000 cells/µL
Sodium	128 mEq/L	135–145 mEq/L
Chloride	97 mEq/L	98–107 mEq/L
Bicarbonate	14 mEq/L	22–28 mEq/L
Anion gap	19	8–16
Blood urea nitrogen	21 mg/dL	7–20 mg/dL
Creatinine	2.23 mg/dL	0.6–1.3 mg/dL
Lactate	2.3 mmol/L	0.5–2.2 mmol/L
Magnesium	1.2 mg/dL	1.7–2.2 mg/dL
Thyroid-stimulating hormone	6.25 µIU/ml	0.4–4.0 µIU/ml
Aspartate aminotransferase	29	10–40 U/L
Alanine aminotransferase	28	7–56 U/L

**Figure 1:**
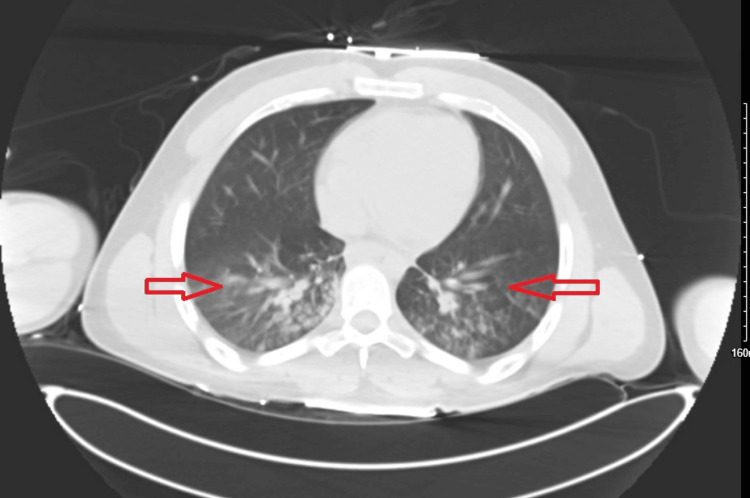
Initial computed tomography showing bilateral opacities and infiltrates suspicious for pneumonia (red arrows).

Also, his pro-B-type natriuretic peptide was elevated to 1,830 pg/mL (reference range = <125 pg/mL) with normal troponins. Transaminases were normal. Several investigations were performed, including a CT of the chest and abdomen, which revealed faint bilateral infiltrates in both lower lung lobes, suspicious for pneumonia (Figure 1).

**Figure 2:**
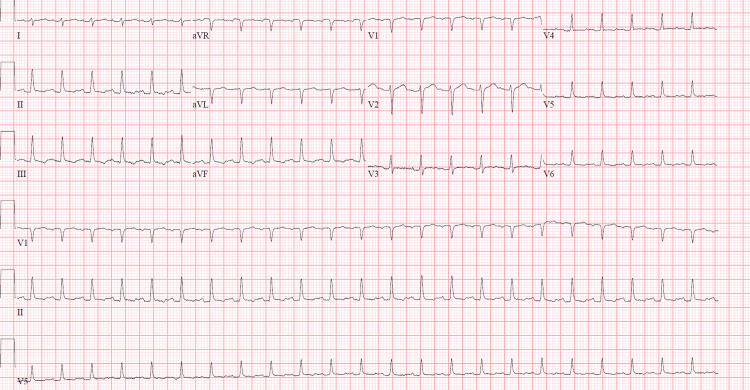
Initial ECG showing low-amplitude QRS complexes and sinus tachycardia.

Respiratory viral panel results were negative for all pathogens except for influenza A/H1 2009, which was positive. The methicillin-resistant *Staphylococcus aureus* DNA nasal probe came back positive. The ECG showed low-voltage QRS complexes with sinus tachycardia (Figure 2).

**Figure 3:**
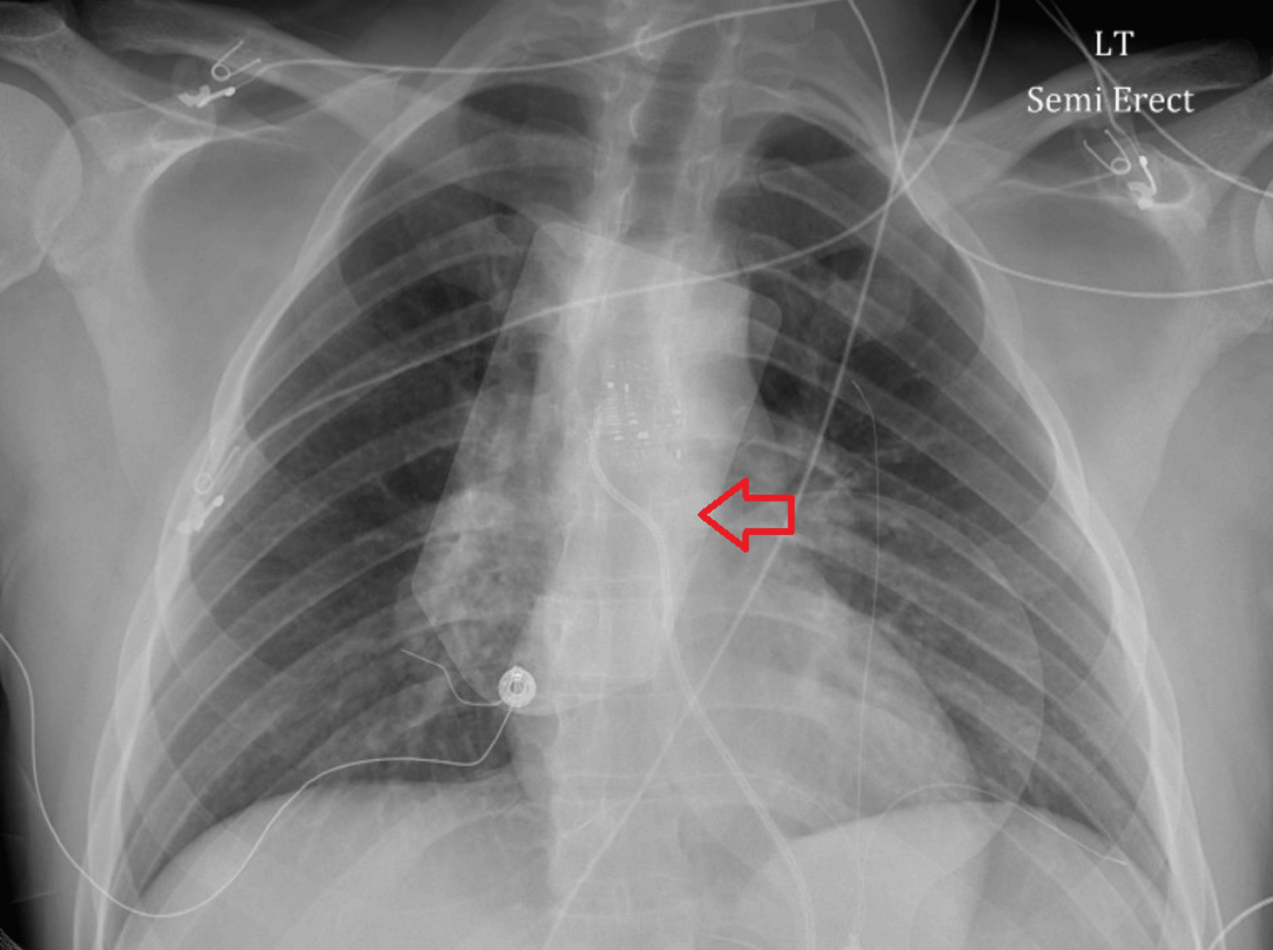
Initial chest X-ray not showing acute cardiopulmonary changes. Also shown with red arrow are the defibrillator pads placed after low ejection fraction was seen.

An echocardiogram revealed a left ventricular ejection fraction of 15-20% with bilateral global systolic dysfunction and normal pericardium. A chest X-ray was performed as part of the initial investigation, which did not show any acute abnormalities (Figure 3).

A CT of the head without contrast did not show any acute cerebral changes. The urine drug screen was negative, and urinalysis revealed waxy casts with increased red blood cells without any evidence of a urinary tract infection.

**Figure 4:**
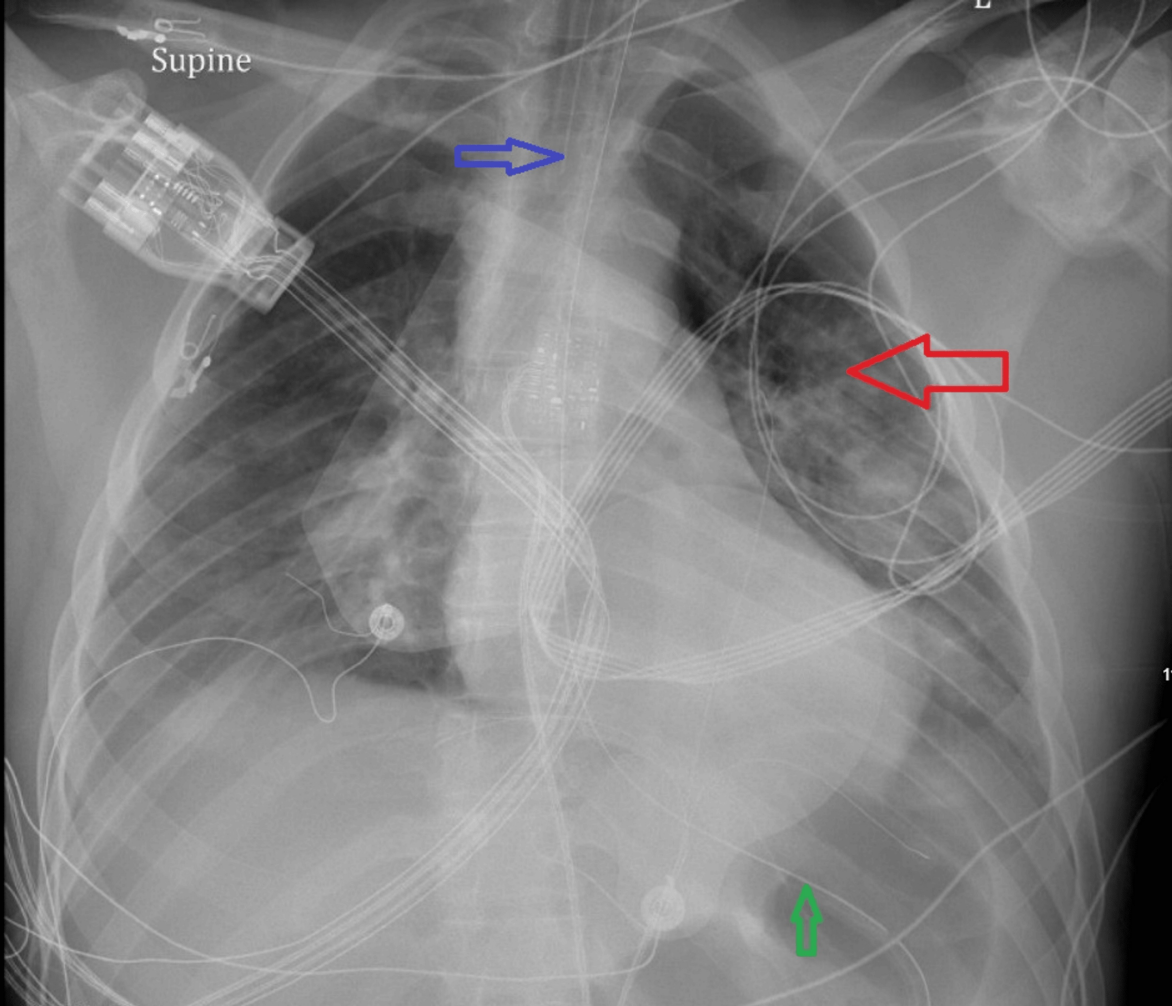
Chest X-ray obtained after endotracheal tube intubation. The red arrow shows prominent infiltrates on the left side. The blue arrow shows the endotracheal tube. The green arrow shows the nasogastric tube.

On day 1, the patient was empirically started on vancomycin q24 hours, piperacillin/tazobactam q6 hours, and acyclovir to cover a broad range of potential infections. Given the patient’s respiratory panel results, oseltamivir was also started. However, the patient’s status deteriorated overnight, and he was intubated for hypoxic respiratory failure and airway protection. He was sedated with Versed and fentanyl infusions to maintain a goal Richmond Agitation-Sedation Scale score of -2 to -3. He subsequently developed acute kidney injury, likely due to acute tubular necrosis. Chest X-ray obtained after endotracheal tube intubation showed adequate tube positioning as well as bilateral infiltrates, more prominent on the left side (Figure 4). This was in contrast with the chest X-ray obtained on arrival in the emergency room, which showed no cardiopulmonary abnormalities. A nasogastric tube was also inserted post-intubation. Acyclovir was later discontinued when a lumbar puncture was not diagnostic of viral meningitis.

On day two, the patient’s hemodynamic status remained unstable, which prompted the initiation of norepinephrine followed by vasopressin for blood pressure support. A left and right heart catheterization was performed, which revealed normal coronary arteries and confirmed biventricular heart failure. Clinical myocarditis was attributed to Influenza A infection. To manage his cardiogenic shock, Impella CP and Impella RP devices were inserted for MCS. Enoxaparin was switched to heparin for anticoagulation. Given the need for higher-level care, the patient was airlifted to another hospital for continued management.

On day four, the right femoral Impella CP was removed, and the femoral site was repaired. The patient was then transitioned to a right axillary Impella 5.5, while the Impella RP was continued for right ventricular support. Vasopressin was discontinued as the patient’s hemodynamics stabilized.

By day five, the Impella RP was successfully removed, and the patient showed significant clinical improvement. He was extubated, and pressor support was stopped. Initially on 4 L oxygen after extubating, he was weaned off to room air the same day. An echocardiogram revealed an improved LVEF of 45%, with the right ventricle mildly dilated but a normal right ventricular ejection fraction.

**Figure 5:**
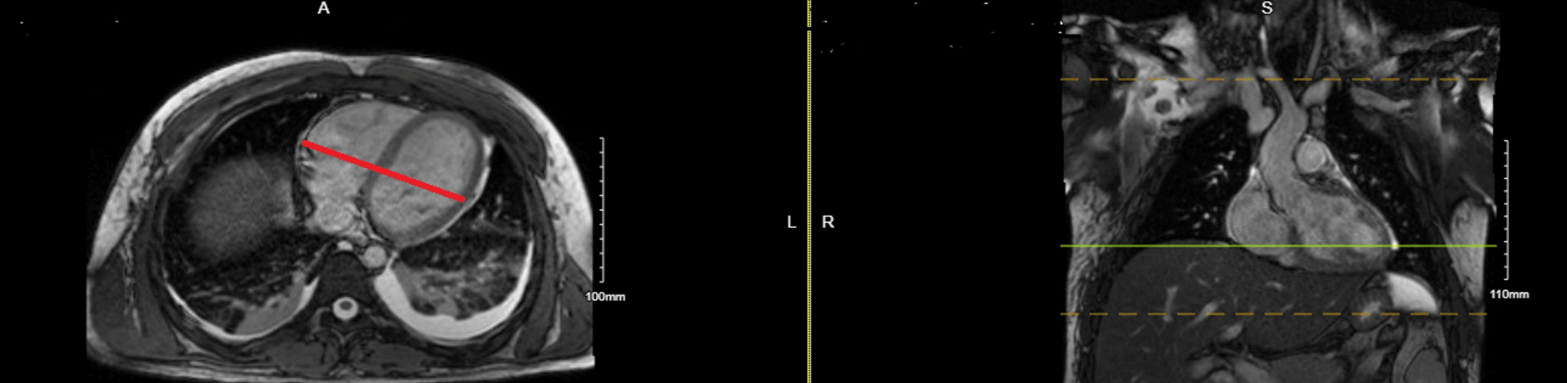
MRI taken after the resolution of the patient’s myocarditis. The image reveals normal left ventricular size, wall thickness, as well as absence of delayed enhancement to suggest infiltrative process.

On day six, a thin liquid diet was initiated after a Speech-Language Pathology evaluation. On day seven, the Impella 5.5 was removed as the patient’s condition continued to improve. By day eight, an MRI showed resolution of myocarditis with an ejection fraction of 65% and a normal left ventricular size (Figure 5), marking significant recovery from his initial illness. The complete timeline of the patient’s hospital course is presented in Table 2, along with a brief description of each hospital day.

**Table 2:** Clinical timeline of the patient’s management.

Day	Clinical events and management
Day 1	• Presented with altered mental status, flu-like symptoms • Vitals: blood pressure, 118/57 mmHg; heart rate, 167 beats/minute; respiratory rate, 30 breaths/minute; temperature, 100.9°F • Pale, diaphoretic, tachycardic • CT chest/abdomen: bilateral lower lobe infiltrates suspicious for pneumonia • CT head: no acute intracranial changes • Lumbar puncture: normal cerebrospinal fluid findings • Echocardiogram: left ventricular ejection fraction (LVEF) 15–20%, global biventricular dysfunction • ECG: low voltage QRS, sinus tachycardia • Positive influenza A, positive methicillin-resistant Staphylococcus aureus culture • Started on vancomycin, piperacillin/tazobactam, acyclovir, oseltamivir • Intubated for hypoxic respiratory failure; developed acute kidney injury (likely acute tubular necrosis) • Nasogastric tube inserted
Day 2	• Persistent unstable hemodynamics; vasopressin and norepinephrine initiated • Cardiac catheterization: normal coronaries, confirmed biventricular failure • Mechanical circulatory support with Impella CP (left ventricle) and Impella RP (right ventricle) • Switched anticoagulation from enoxaparin to heparin • Airlifted to Baptist East for higher-level care
Day 4	• Removal of femoral Impella CP, femoral site repaired • Transitioned to right axillary Impella 5.5; continued Impella RP • Vasopressin discontinued (improved hemodynamics)
Day 5	• Impella RP removed • Clinical improvement noted: extubated, pressors discontinued • Oxygen therapy weaned from 4 L to room air • Echocardiogram: improved LVEF 45%, mild right ventricular (RV) dilation, normal RV function
Day 6	• Initiated on a thin liquid diet after Speech-Language Pathology evaluation
Day 7	• Impella 5.5 removed due to continued clinical stability and improvement
Day 8	• MRI: myocarditis resolved, normalized LVEF of 65%, normal left ventricular size

## Discussion

Influenza, a viral respiratory pathogen, is associated with clinical myocarditis in nearly 10% of infections worldwide [2]. Fulminant myocarditis due to influenza is managed through a variety of MCS systems, including Impella, extracorporeal membrane oxygenation, and intra-aortic balloon pumps, and these devices have shown impressive outcomes [3,4]. Our case demonstrates the successful use of bilateral Impella devices in a patient with cardiogenic shock due to myocarditis. Several studies have shown promising outcomes with the use of Impella for fulminant myocarditis. In a study of the Global cVAD Registry, which included 34 patients with myocarditis treated with Impella devices, 62% of patients were discharged with an improved mean left ventricular ejection fraction [4]. This improvement is consistent with our case, as the left ventricular ejection fraction improved from 20% to 65%.

In our case, there appears to be a considerable genetic predisposition to the development of influenza-induced myocarditis, considering the patient’s familial history of myocarditis affecting both the mother and grandfather. This is supported by the findings from a study by Belkaya et al., which demonstrated that patients with acute myocarditis were enriched with rare variants in genes associated with inherited cardiomyopathies, suggesting a genetic predisposition [5]. Despite the likely genetic predisposition, the acute presentation of this patient after a viral infection favors the diagnosis of influenza-induced myocarditis, as acute myocarditis is more likely to present with an acute or fulminant onset compared to genetic cardiomyopathy [6].

According to the American Heart Association, for diagnosing myocarditis, a patient must have at least one clinical presentation criterion (such as chest pain, heart failure, arrhythmia, or shock) plus at least one diagnostic criterion (such as abnormal ECG, elevated troponin, ventricular dysfunction, or MRI findings); however, if no clinical presentation criteria are present, at least two diagnostic criteria are required. [7] In addition, literature has also established the role of urine metabolomic profiling in determining distinct metabolic signatures associated with acute myocarditis, including changes in amino acid, lipid, carbohydrate, and nucleotide metabolism. In a study using high-throughput metabolomics, 19 urinary metabolites were identified as potential biomarkers for acute myocarditis. However, these findings are preliminary and require further studies before clinical use [8].

Our patient demonstrated significant improvement in cardiac function and a remarkable recovery of ejection fraction after receiving timely treatment with oseltamivir and MCS with Impella. This case reinforces the importance of early recognition and successful use of Impella devices in young patients presenting with cardiogenic shock due to fulminant myocarditis. Additionally, it also highlights the need for further research to explore the potential genetic link between viral infections and myocarditis, which could help identify at-risk individuals and improve prognosis.

The limitations of this case report include having a single case instead of a case series. Given the recent occurence of this case, we lack the availability of extensive patient follow-up, including testing for a genetic predisposition.

## Conclusions

Fulminant myocarditis is a rare but potentially fatal side effect of influenza that, even in young, healthy people, can result in acute biventricular failure and cardiogenic shock. To improve results, this severe cardiac dysfunction needs to be identified quickly and aggressively managed. MCS devices, especially the Impella system, have become essential for stabilizing patients with fulminant myocarditis leading to systolic heart failure. To effectively unload the heart, maintain systemic perfusion, and facilitate myocardial recovery, Impella devices offer targeted left and right ventricular support. In this case, fulminant influenza struck a 24-year-old man who had no previous cardiovascular risk factors and led to myocarditis that caused cardiogenic shock and severe biventricular failure. Bilateral Impella support was started as soon as possible to stabilize the patient, which resulted in a notable improvement in cardiac function. Within days, the left ventricular ejection fraction rose dramatically from 15-20% to 65%, highlighting the possibility of myocardial recovery with proper support. The severity of influenza A is demonstrated in this case, underscoring the vital need for clinicians to identify early indicators of cardiac involvement in influenza infections. For patients with fulminant myocarditis, early diagnosis and the start of mechanical support with Impella devices can save lives, prevent irreversible cardiac damage, and increase survival rates. Optimizing results in these high-risk situations still requires greater awareness and early intervention.
